# CD47 Deficiency in Mice Exacerbates Chronic Fatty Diet-Induced Steatohepatitis Through Its Role in Regulating Hepatic Inflammation and Lipid Metabolism

**DOI:** 10.3389/fimmu.2020.00148

**Published:** 2020-02-25

**Authors:** Hui-Chao Tao, Ke-Xin Chen, Xue Wang, Bo Chen, Wai-Ou Zhao, Yang Zheng, Yong-Guang Yang

**Affiliations:** ^1^Key Laboratory of Organ Regeneration and Transplantation of the Ministry of Education, Institute of Immunology, The First Hospital, Jilin University, Changchun, China; ^2^National–Local Joint Engineering Laboratory of Animal Models for Human Diseases, Changchun, China; ^3^Cardiovascular Center, The First Hospital, Jilin University, Changchun, China; ^4^Columbia Center for Translational Immunology, Department of Medicine, Columbia University College of Physicians and Surgeons, New York, NY, United States

**Keywords:** non-alcoholic steatohepatitis—NASH, lipid metabolism, inflammation, cd47, fatty diet

## Abstract

Inflammation is one of the hallmarks of non-alcoholic steatohepatitis. CD47 is a widely expressed transmembrane protein that signals through inhibitory receptor signal regulatory protein α (SIRPα) to inhibit macrophage activation and phagocytosis. In this study, we sought to investigate the role of CD47 in hepatosteatosis and fibrosis induced by a chronic high-fat diet (HFD), by comparing disease development in wild-type (WT) and CD47KO mice fed HFD for 40 weeks. The HFD induced remarkably more severe hepatic steatosis and fibrosis but less body weight gain and less subcutaneous fat accumulation in CD47KO mice compared to WT mice. Liver tissues from HFD-fed CD47KO mice exhibited enhanced inflammation characterized by increased proinflammatory cytokine production and increased nuclear factor-κB (NF-κB) activation compared to similarly fed WT mice. Although higher expression of apolipoproteins was observed in CD47KO mice compared to WT mice under a low-fat diet (LFD), HFD-fed WT and CD47KO mice showed comparably prominent downregulation of these apolipoprotein genes, suggesting that the marked difference observed in lipid accumulation and hepatosteatosis between these mice cannot be explained by changes in apolipoproteins. Like apolipoproteins, sirtuin 1 (SIRT1) and peroxisome proliferator activated receptor alpha (PPARα), which are involved in regulation of both lipid metabolism and inflammation, were more highly expressed in CD47KO than WT mice under LFD but more severely suppressed in CD47KO than in WT mice under HFD. Taken together, our results indicate that CD47 plays a significant role in the pathogenesis of HFD-induced hepatosteatosis and fibrosis through its role in regulation of inflammation and lipid metabolism.

## Introduction

Non-alcoholic steatohepatitis (NASH) is the severe form of non-alcoholic fatty liver disease (NAFLD) characterized by excessive fat accumulation and inflammation in the liver (1, 2). The pathological features of NASH include hepatic injury, inflammation, and excessive production of extracellular matrix (ECM) proteins, particularly collagen. Activation of proinflammatory factors, such as the transcription factor nuclear factor-κB (NF-κB), a key regulator of inflammation, plays an important role in the progression of NASH (3). Although the mechanism for linking inflammation and fibrosis has not yet been fully clarified, liver damage–induced activation of resident inflammatory cells and recruitment of monocytes/macrophages have been reported to promote the development of fibrosis (4). There is increasing evidence to indicate that monocyte/macrophage infiltration results in elevated chemokine production and fibrosis-associated angiogenesis and that increased levels of chemokine C-C motif ligand 2 (CCL2) and its receptor (CCR2) in turn further aggravate monocyte/macrophage infiltration to the site of injury (5–7).

CD47 is a ubiquitously expressed transmembrane protein that serves as a ligand for signal regulatory protein α (SIRPα) and as a signaling receptor for thrombospondin 1 (TSP1) (8). CD47–SIRPα pathway activation provides inhibitory signaling in monocytes and macrophages, attenuating phagocytosis and inflammation (9–11). This pathway may become even more important in controlling macrophage activation under inflammatory conditions (12) and after allogeneic or xenogeneic transplantation (10, 13–15). Although the role of CD47–SIRPα signaling in inhibition of inflammatory responses has been well-documented in numerous models, the involvement of this pathway in fatty acid–induced inflammatory responses is relatively unexplored. A recent report shows that CD47 deficiency may attenuate obesity and lipid accumulation in the liver of mice fed a high-fat diet (HFD) for 16 weeks (16). Because inflammation is a key factor driving the progression of NASH, it is imperative to understand the precise role of CD47 in the pathogenesis of NASH.

In this study, we sought to investigate the role of CD47 in fatty diet consumption–induced NASH by comparing hepatic lipid metabolism, steatosis, fibrosis, and inflammation between wild-type (WT) and CD47KO mice chronically fed HFD for 40 weeks. We show that CD47 deficiency significantly attenuated body weight gains and subcutaneous fat accumulation, while severely worsening hepatic steatosis and fibrosis induced by prolonged HFD consumption. CD47 deficiency also elevated monocyte/macrophage infiltration, inflammation, and NF-κB activation in the liver of mice chronically fed HFD. Furthermore, HFD feeding of CD47KO mice induced more significant downregulation of sirtuin 1 (SIRT1) and peroxisome proliferator activated receptor alpha (PPARα), which are involved in regulation of both lipid metabolism and inflammation. These results provide direct evidence for a significant role of CD47 in regulation of hepatic inflammation and lipid metabolism and in the development of NASH induced by chronic HFD consumption.

## Methods

### Animal Experiments

WT C57BL/6 (B6) mice and CD47KO mice on the B6 background were originally purchased from Charles River (Beijing, China) and the Jackson laboratory, respectively, and were bred in our specific pathogen–free (SPF) mouse facility for more than a year before use in this study. Eight-week-old male mice were fed HFD (45% kcal from fat; D12451, XieTong Pharmaceutical Bio-Engineering, China; Table S1) or a low-fat diet (LFD) (10% kcal from fat; D12450B; XieTong Pharmaceutical Bio-Engineering, China) for 40 weeks. Body weight was measured monthly at the same time. At the indicated times, mice were sacrificed, and blood and liver tissues were collected for analysis as detailed below. Protocols involving animal experiments were approved by the Subcommittee on Research Animal Care of the First Hospital of Jilin University, and all of the experiments were performed in accordance with the protocols.

### Analysis of Serum Chemistry

Mouse serum lipids, including high-density lipoprotein cholesterol (HDL-C), low-density lipoprotein cholesterol (LDL-C), and total cholesterol (TC) were examined by Cholesterol E kit (439–17501, Wako) and HDL-Cholesterol E kit (431-52501, Wako). Serum alanine aminotransferase (ALT) levels were measured by an ELISA kit for ALT with triplicates for each sample (SEA207Mu, USCN, China) according to the manufacturer's instructions.

### Analysis of Lipid Accumulation in Liver

Liver triglyceride (TG) content was measured using a Triglyceride Assay Kit (Abcam, ab65336) according to the manufacturer's instructions. Lipid droplets were assessed by Oil Red O staining. Fresh frozen liver tissues were cryostat-sectioned at 5 μm and fixed in 4% PFA for 10 min at room temperature (RT). Slides were blotted in 60% isopropyl alcohol for 10 min and then stained with filtered Oil Red O for 20 min at RT. Slides were rinsed once with distilled water, mounted, and sealed with nail polish.

### Quantitative Real-Time PCR

Total RNA was extracted with an Axyprep multisource RNA miniprep kit (Axygen, America), and cDNA was synthesized using TransScript First-Strand cDNA Synthesis SuperMix (TransGen Biotech, Beijing). Quantitative real-time PCR was performed using a SYBR Green kit (TransGen Biotech, Beijing) with a StepOnePlus real-time PCR system (ABI), and the primer sets used for MTTP, ApoA1, ApoB, ApoC2, CD31, TGFβ, TSP1, VEGFR1, IL-1β, TNFα, IL-6, IL-10, CCL2, PPARα, SIRT1, and β-actin (Sangon Biotech, Shanghai) are listed in Table 1. Relative gene expression was measured with triplicates for each sample and normalized to β-actin.

**Table 1:** Primer sequences for qPCR.

**Mouse genes**	**Forward (5′-3′)**	**Reverse (5′-3′)**
β-actin	TTCAACACCCCAGCCATG	CCTCGTAGATGGGCACAGT
ApoA1	CTTGGCACGTATGGCAGCA	CCAGAAGTCCCGAGTCAATGG
ApoB	ACGGGCAATGAAGACCACAC	CGGGAGCGACACCATTTACAA
ApoC2	ATGGGGTCTCGGTTCTTCCT	GTCTTCTGGTACAGGTCTTTGG
CD31	AGGCTTGCATAGAGCTCCAG	TTCTTGGTTTCCAGCTATGG
TSP1	TTGCCAGCGTTGCCA	TCTGCAGCACCCCCTGAA
TGFβ	CCACCTGCAAGACCATCGAC	CTGGCGAGCCTTAGTTTGGAC
TNFα	GTCCTGCTCTACGTGACGAG	TGCAGATGTACCTTGGAGAGTA
MTTP	ATACAAGCTCACGTACTCCACT	TCTCTGTTGACCCGCATTTTC
CCL2	GAAGGAATGGGTCCAGACAT	ACGGGTCAACTTCACATTCA
VEGFR1	CGGAAGGAAGACAGCTCATC	CTTCACGCGACAGGTGTAGA
IL-1β	TGGACCTTCCAGGATGAGGACA	GTTCATCTCGGAGCCTGTAGTG
IL-6	CCTCTGGTCTTCTGGAGTACC	ACTCCTTCTGTGACTCCAGC
IL-10	ATAACTGCACCCACTTCCCA	GGGCATCACTTCTACCAGGT
PPARα	TACTGCCGTTTTCACAAGTGC	AGGTCGTGTTCACAGGTAAGA
SIRT1	ATGACGCTGTGGCAGATTGTT	CCGCAAGGCGAGCATAGAT

### Immunoblotting

Total lysate was extracted from liver tissues on ice with radioimmunoprecipitation assay (RIPA) lysis buffer and a protease/phosphatase inhibitor cocktail (Roche Diagnostics). The homogenates were centrifuged at 10,000 × g for 20 min at 4°C. The protein amounts were assessed by the BCA method (Thermo, #23225). Phosphorylated NF-κB p65 (pNF-κB, Ser563) and β-actin in the total lysate were detected by anti-phos-NF-κB (Affinity, AF5006) and anti-β-actin (Biolegend, 622102) antibodies, respectively. The proteins were visualized with a Super Signal West Pico-Chemiluminescent Substrate (Tanon, 180-501) according to the manufacturer's protocol.

### Histology, Immunohistochemistry, and Immunofluorescence Analysis

Tissues were harvested and fixed with 10% formalin and embedded in paraffin. Serial sections (4 μm) were prepared and stained with H&E and immunohistochemistry (IHC). For IHC, tissue sections were subjected to antigen retrieval and incubation with primary antibody against CCL2 (BF0556, Affinity), TGF-β (AF1027, Affinity), IL-1β (AF5103, Affinity), IL-6 (DF6087, Affinity), or IL-10 (DF6894, Affinity), followed by incubation with a peroxidase-conjugated goat anti-rabbit IgG (KIT-9706, Maixin-Bio) secondary antibody, and the immunoreactivity was detected with an UltraSensitive™ Streptavidin-Peroxidase Kit (KIT-9710, Mai Xin, China) according to the manufacturer's protocol. The collagen content was assessed by Sirius Red (365548, Sigma). For immunofluorescence (IF), cryosections (4 μm) were prepared from freshly frozen liver tissues and incubated with antibodies specific for mouse CD68 (137005, Biolegend), CD31-antibody (102432, Biolegend), TIE2 (124008, Biolegend), or phos-NF-κB (Affinity, AF5006). Photomicrographs were produced using a confocal microscope and corresponding software (Zeiss). Image Pro Plus 6.0 software was used for quantitative analysis.

### Statistical Analysis

All data are presented as mean ± SD, and the statistical analysis was performed using Prism 6 (GraphPad Software). One-way ANOVA was used for comparing multiple groups, while the *t*-test was used for comparing two groups. Differences with p < 0.05 were considered statistically significant.

## Results

### HFD Induces Severe Hepatomegaly and Liver Injury but Less Subcutaneous Fat Accumulation in CD47-Deficient Mice

**Figure 1:**
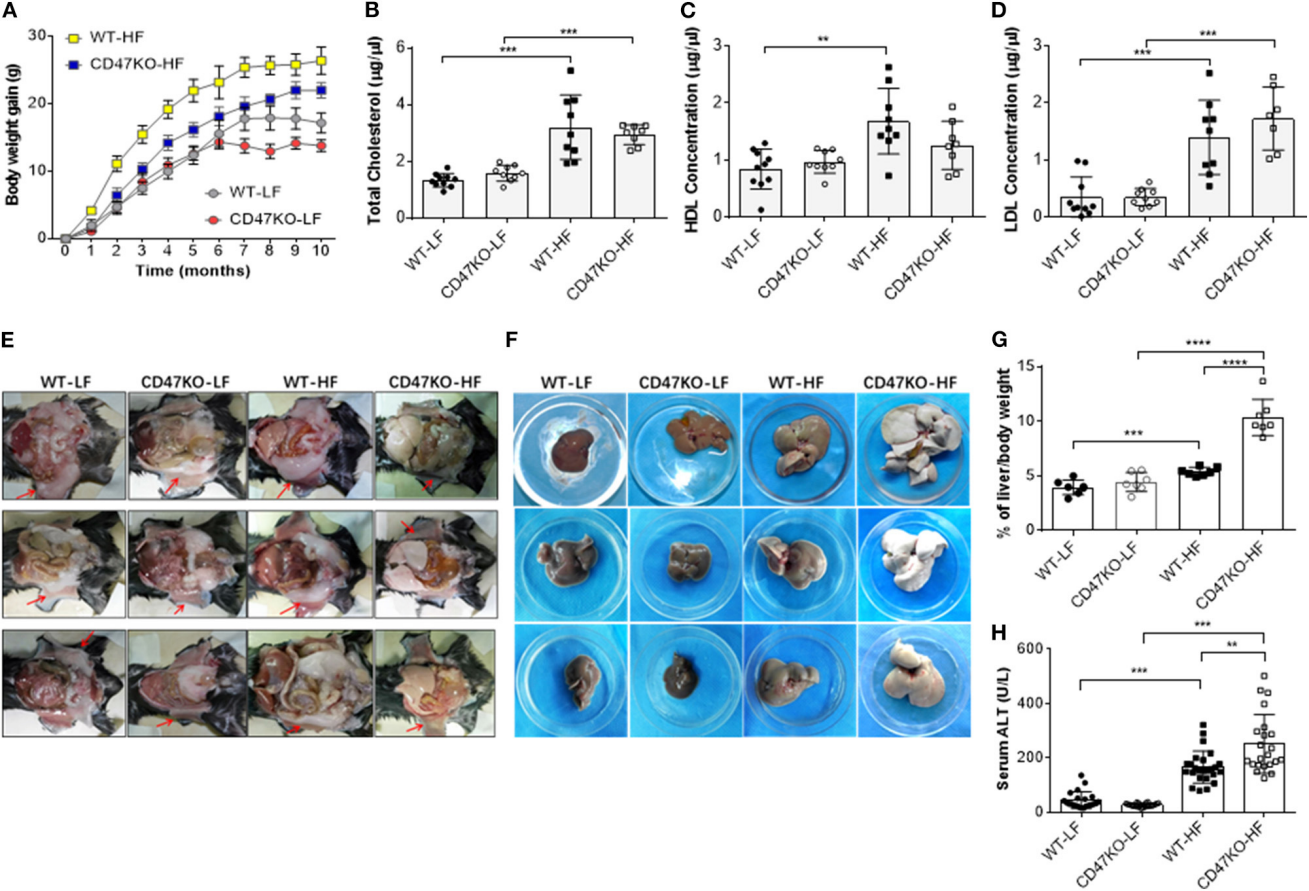
A high-fat diet (HFD) induces more severe liver injury in CD47KO mice. **(A)** Net body weight gains (*n* = 7 mice/group). A representative of two independent experiments is shown. **(B–D)** Serum levels of total cholesterol **(B)**, high-density lipoprotein cholesterol (HDL) **(C)**, and low-density lipoprotein cholesterol (LDL) **(D)** measured at the end of the experiments (*n* = 7–10). Shown are results from a representative of two independent experiments. **(E,F)** Autopsy showing subcutaneous fat (**E**, arrowhead) and hepatomegaly **(F)**. **(G)** Ratios (%) of liver to body weight (*n* = 7 mice/group). **(H)** Serum alanine aminotransferase (ALT) levels (*n* = 22–25; combined from four independent experiments). Data are presented as mean ± SD. ^**^p < 0.01; ^***^P < 0.001; and ^****^P < 0.0001.

In order to assess the role of CD47 in the development of fatty liver and obesity, we compared hepatic steatosis and subcutaneous fat accumulation in WT and CD47KO mice that were chronically fed LFD (10% kcal from fat) or HFD (45% kcal from fat) for 40 weeks. HFD-fed CD47KO mice showed a significantly delayed and lower increase in body weight compared to similarly HFD-fed WT mice (Figure 1A). When fed LFD, WT and CD47KO mice showed a similar increase in body weight within the first 6 months; then the CD47KO mice stopped gaining weight immediately, which was 1 month earlier than the WT mice (Figure 1A). HFD also induced a significant increase in total serum cholesterol (TC; Figure 1B) and LDL-C (Figure 1D) in both WT and CD47KO mice. However, a significant increase in HDL-C was only detected in HFD-fed WT mice (Figure 1C). Furthermore, HFD induced prominent subcutaneous fat accumulation (Figure 1E) and hepatomegaly (Figure 1F) in WT mice. Surprisingly, HFD-fed CD47KO mice showed almost no subcutaneous fat accumulation but extremely severe hepatomegaly compared to HFD-fed WT mice (Figures 1E,F). In HFD-fed CD47KO mice, the liver/body weight ratio was significantly increased (by over two-fold) compared to mice in all other groups (liver/body weight ratios were comparable among LFD-fed WT and CD47KO mice, and HFD-fed WT mice; Figure 1G). Moreover, HFD also resulted in more severe liver injury in CD47KO mice than in WT mice, as shown by ALT serum activity (Figure 1H). These data indicate that CD47 deficiency augments HFD-induced hepatomegaly and liver injury but suppresses subcutaneous fat accumulation and the associated increase in body weight, suggesting that CD47 plays an important role in lipid metabolism and/or distribution.

### CD47 Deficiency in Mice Promotes HFD-Induced Lipid Accumulation in Liver but Not Downregulation of Hepatic Apolipoproteins

**Figure 2:**
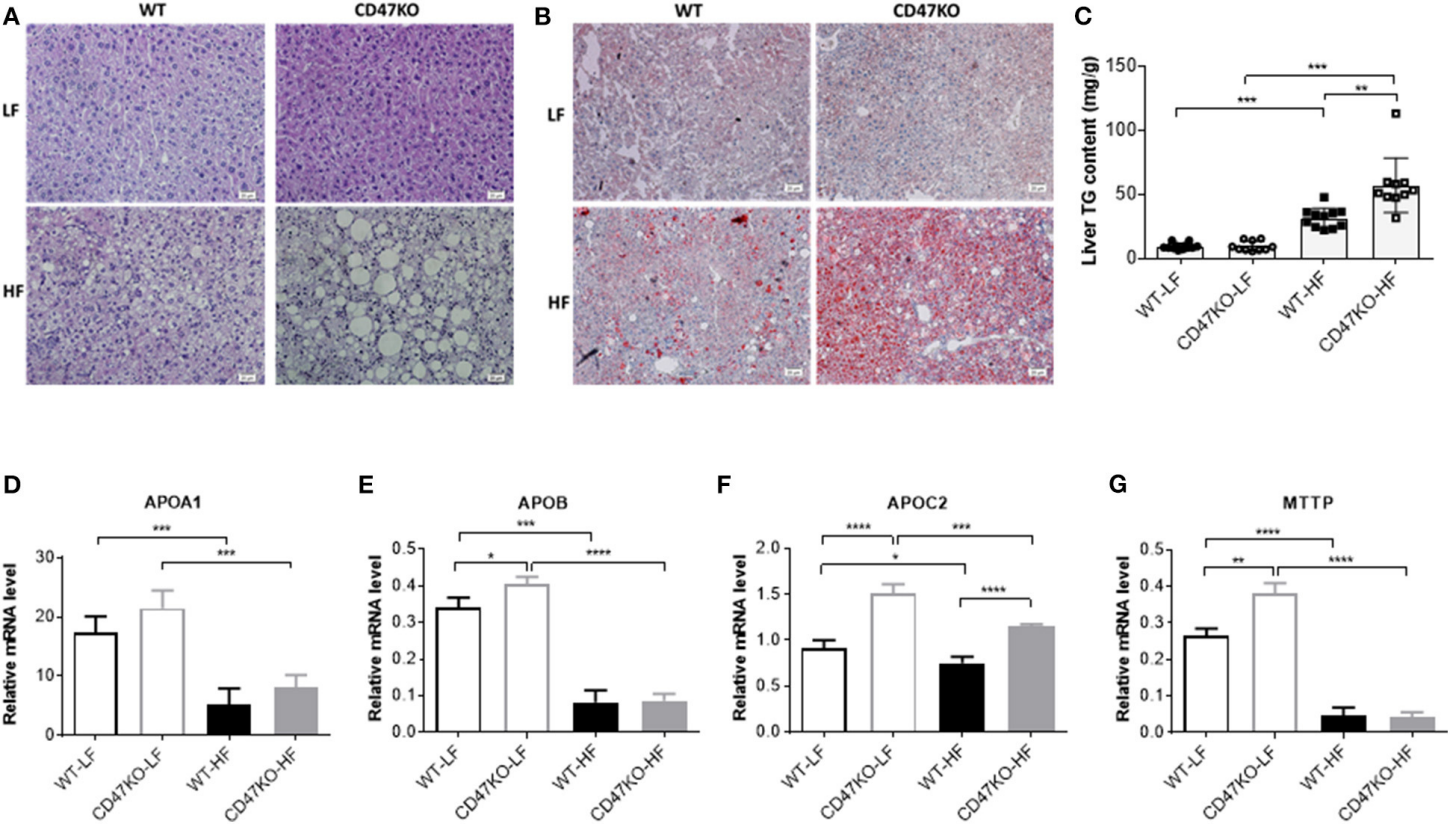
Lipid accumulation and apolipoprotein gene expression in livers from wild-type (WT) and CD47KO mice fed a low-fat diet (LFD) or HFD. **(A,B)** Representative image of H&E **(A)** and Oil Red O (**B**; lipid droplet accumulation is shown in red) staining of liver tissues from WT and CD47KO mice fed LFD or HFD (three mice per group). Scale bar represents 20 μm. **(C)** Triglyceride (TG) content in liver extracts from the indicated groups of mice (*n* = 10–12 mice/group; combined from two independent experiments). **(D–G)** Expression Levels of APOA1 **(D)**, APOB **(E)**, APOC2 **(F)**, and MTTP, which were determined by quantitative real-time PCR and normalized to β-actin (*n* = 4 per group). Data are presented as mean ± SD. ^*^p < 0.05; ^**^p < 0.01; ^***^P < 0.001; and ^****^P < 0.0001.

Histological analysis was performed to determine whether CD47 deficiency may promote the development of fatty liver disease. In accordance with the more severe hepatomegaly and liver injury in HFD-fed CD47KO mice (Figures 1F–H), H&E (Figure 2A) and Oil Red O staining (Figure 2B) revealed that HFD induced more severe hepatocyte ballooning and excessive lipid accumulation in CD47KO mice compared to WT mice, whereas no significant difference was detected between LFD-fed CD47KO and WT mice. Although HFD consistently induced a significant elevation of liver TGs in both WT and CD47KO mice compared to LFD-fed mice, the magnitude of the elevation was significantly less pronounced in WT than CD47KO mice (Figure 2C). Apolipoproteins are involved in the crosstalk between adipose tissue and the liver (17). Because apolipoproteins play important roles in the development of obesity and hepatosteatosis, and their expression can be regulated by liver injury and inflammation (18), we measured the levels of apolipoprotein mRNAs in liver tissues by real-time PCR. Although WT and CD47KO mice fed LFD had comparable expression of APOA1, the levels of APOB, APOC2, and MTTP were significantly higher in the latter group (Figures 2D–G). HFD induced a significant downregulation of all these apolipoproteins in both WT and CD47KO mice (Figures 2D–G). HFD-fed WT and CD47KO mice showed comparably prominent downregulation of these apolipoprotein genes, with the exception of APOC2, which was downregulated to a lesser extent than other apolipoproteins in HFD-fed mice and expressed at a higher level in CD47KO than in WT mice. These data suggest that changes in apolipoproteins cannot explain the observed differences in lipid accumulation and hepatosteatosis between HFD-fed WT and CD47KO mice.

### CD47 Deficiency in Mice Promotes Development of HFD-Induced Liver Fibrosis

**Figure 3:**
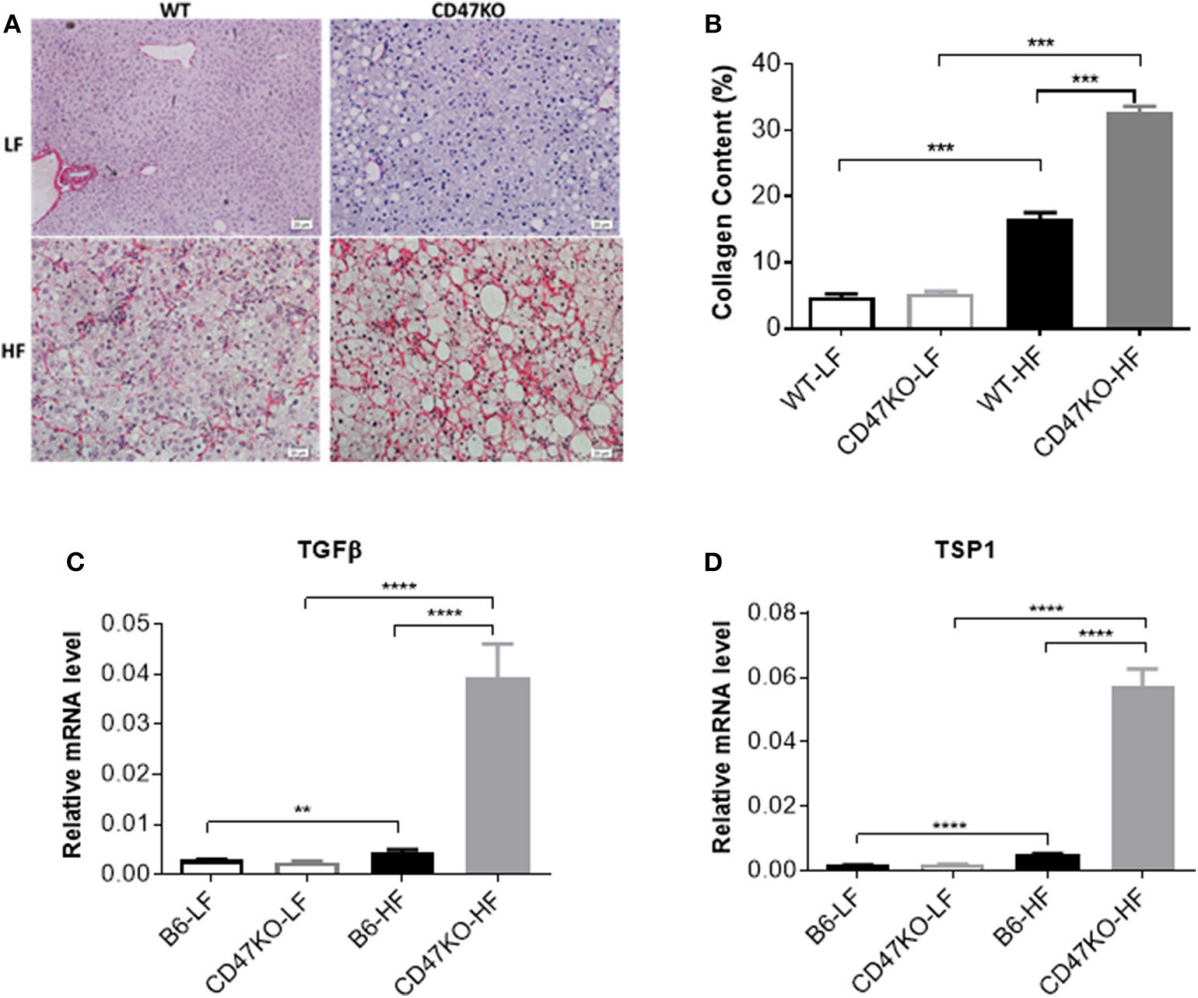
Liver fibrosis in WT and CD47KO mice fed LFD or HFD. **(A)** Liver fibrosis determined by Sirius Red staining. Three samples per group were examined, and representative images are shown. **(B)** Collagen content determined by counting positive areas in six randomly selected fields using Image Pro Plus 6.0 software. Data from a representative of three independent samples are shown. **(C,D)** Relative mRNA expression levels of TGFβ **(C)** and thrombospondin 1 (TSP1) **(D)**, which were determined by quantitative real-time PCR and normalized to β-actin (*n* = 4 per group). Data are presented as mean ± SD. ^**^p < 0.01; ^***^P < 0.001; and ^****^P < 0.0001.

Chronic hepatic injury and subsequent inflammation enhance collagen production, resulting in progression of liver fibrosis (19, 20). Sirius Red staining revealed that HFD-induced fibrosis, as shown by increased fibrillar collagen deposition, was significantly more severe in CD47KO than in WT mice (Figure 3A). Consistently, liver tissues from HFD-fed CD47KO mice displayed significantly higher collagen content (Figure 3B) and upregulated gene expression of pro-fibrotic factor TGFβ (Figure 3C and Figure S1) and TSP1 (Figure 3D), a major physiologic activator of latent TGFβ (8).

### HFD Induces Robust Angiogenesis With Upregulation of VEGFR1 and CD31 in the Liver of CD47KO Mice

**Figure 4:**
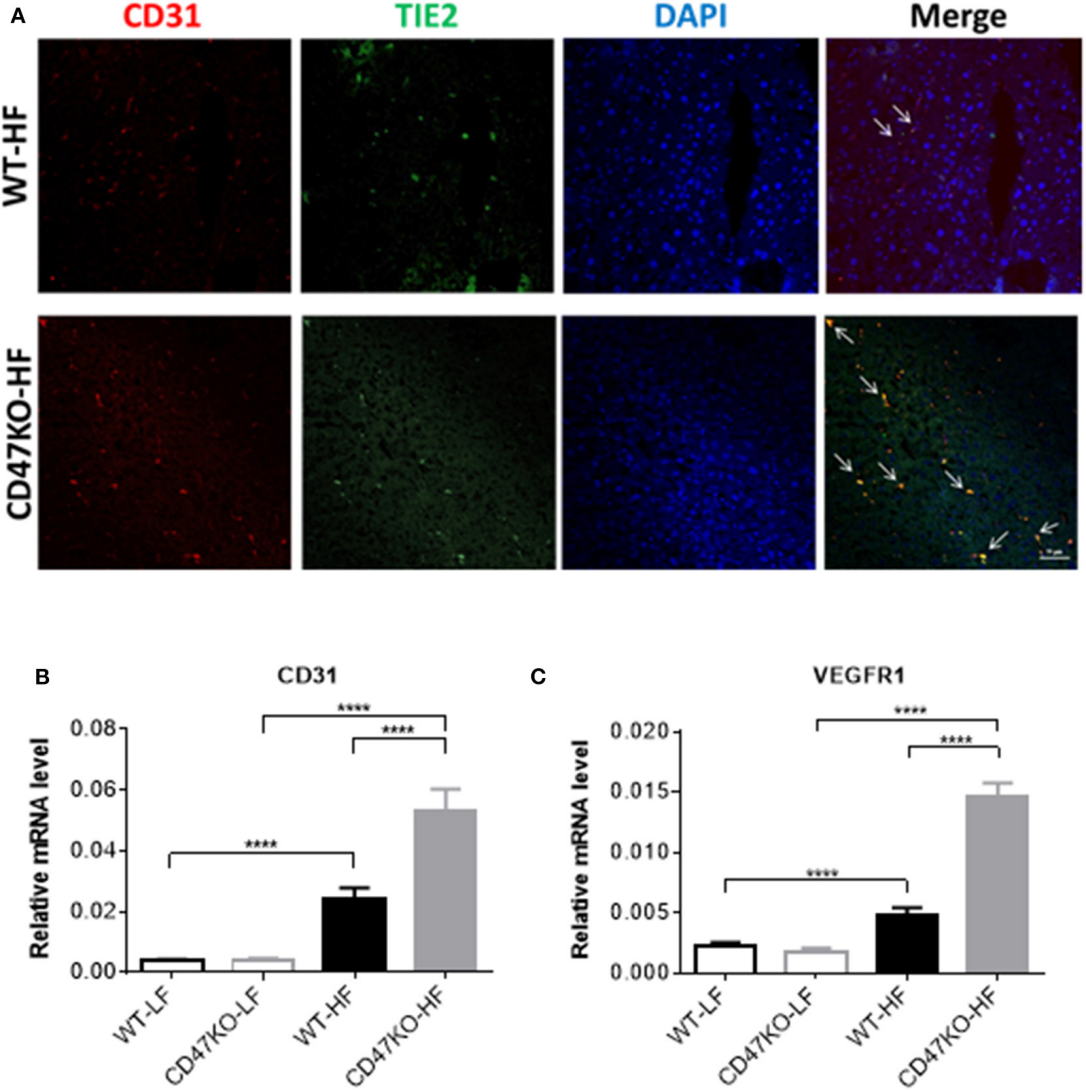
CD47 deficiency promotes HFD-induced angiogenesis with upregulation of CD31 and VEGFR in the liver. **(A)** Representative images of liver sections stained for CD31 (red), TIE2 (green), and DAPI. Merged images are shown in the last column (CD31+TIE2+ cells are indicated by arrows). Data from a representative of three samples are shown. Scale bar represents 50 μm. **(B,C)** Relative mRNA expression levels of CD31 **(B)** and VEGFR1 **(C)** quantified by real-time PCR and normalized to β-actin (*n* = 4 per group). Data are presented as mean ± SD. ^****^P < 0.0001.

Progression of liver fibrosis is accompanied by pathological angiogenesis from the early stages (21, 22). To determine the role of CD47 in fibrotic-associated angiogenesis, we measured microvessel density (MVD) by staining with endothelial markers CD31 and TIE2. As shown in Figure 4A, there was a marked increase of MVD in liver tissues from HFD-fed CD47KO mice compared to similarly fed WT mice. Furthermore, liver tissues from HFD-fed CD47KO mice also had significantly higher levels of CD31 and VEGFR1 gene expression compared to WT mice (Figures 4A–C). These results indicate that CD47 deficiency may promote HFD-induced collagen production and pro-fibrotic factor expression and thus promote fibrosis and the associated abnormal enhancement of angiogenesis.

### CD47 Deficiency in Mice Enhances HFD-Induced Inflammation, Monocyte/Macrophage Infiltration, and NF-κB Activation in the Liver

**Figure 5:**
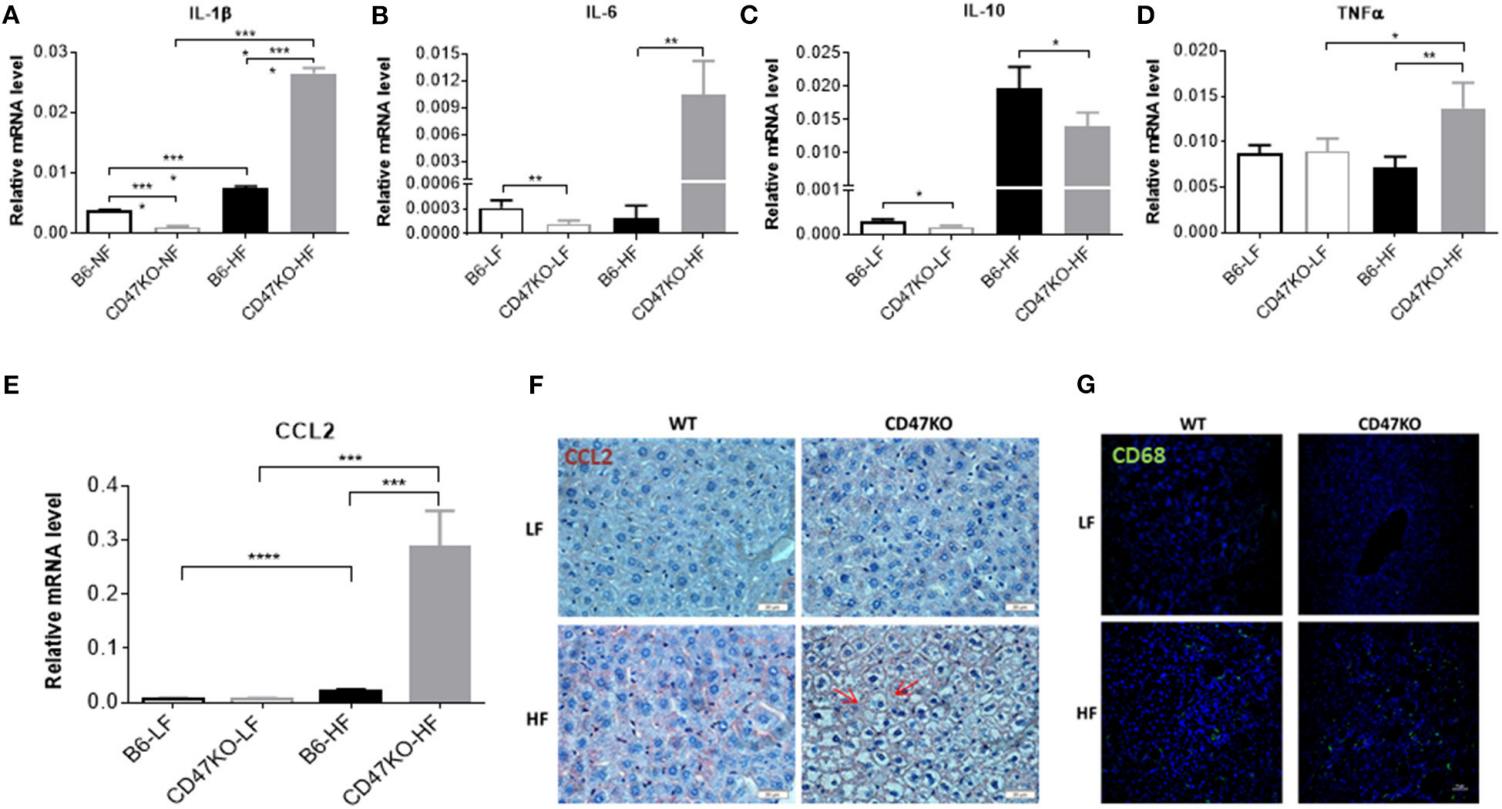
CD47 deficiency enhances HFD-induced inflammation and monocyte/macrophage infiltration in the liver. **(A–E)** Relative mRNA levels of IL-1β **(A)**, IL-6 **(B)**, IL-10 **(C)**, TNFα **(D)**, and C-C motif ligand 2 (CCL2) **(E)**. Gene expression levels were determined by quantitative real-time PCR and normalized to β-actin (*n* = 4 per group). Data are presented as mean ± SD. ^*^p < 0.05; ^**^p < 0.01; ^***^P < 0.001; and ^****^P < 0.0001. **(F)** Liver sections were immunohistochemistry (IHC)-stained for CCL2. Six samples per group were examined, and representative images are shown (scale bar represents 20 μm). **(G)** Liver sections were immunofluorescence-stained by anti-CD68 (green) plus DAPI (blue). Four samples per group were examined, and representative images are shown (scale bar represents 50 μm).

Chronic hepatic injury contributes to activation and recruitment of inflammatory cells such as monocytes and macrophages (23). Therefore, we compared the levels of proinflammatory (IL-1β, IL-6, and TNFα) and anti-inflammatory (IL-10) cytokines in liver tissue between WT and CD47KO mice that were chronically fed HFD or LFD. Compared to LFD-fed animals, liver tissues from HFD-fed CD47KO mice showed markedly increased production of all three pro-inflammatory cytokines, whereas only a moderate increase in IL-1β was detected in liver tissues from HFD-fed WT mice (Figures 5A,B,D and Figures S2A,B). However, the level of IL-10 gene expression was significantly higher in liver tissue from HFD-fed WT mice than in liver tissue from HFD-fed CD47KO mice (Figure 5C and Figure S2C). Furthermore, liver tissue from HFD-fed CD47KO mice displayed a more significant increase in production of CCL2 (Figures 5E,F), which was associated with more severe infiltration by CD68+ monocytes/macrophages (Figure 5G), compared to HFD-fed WT mice.

**Figure 6:**
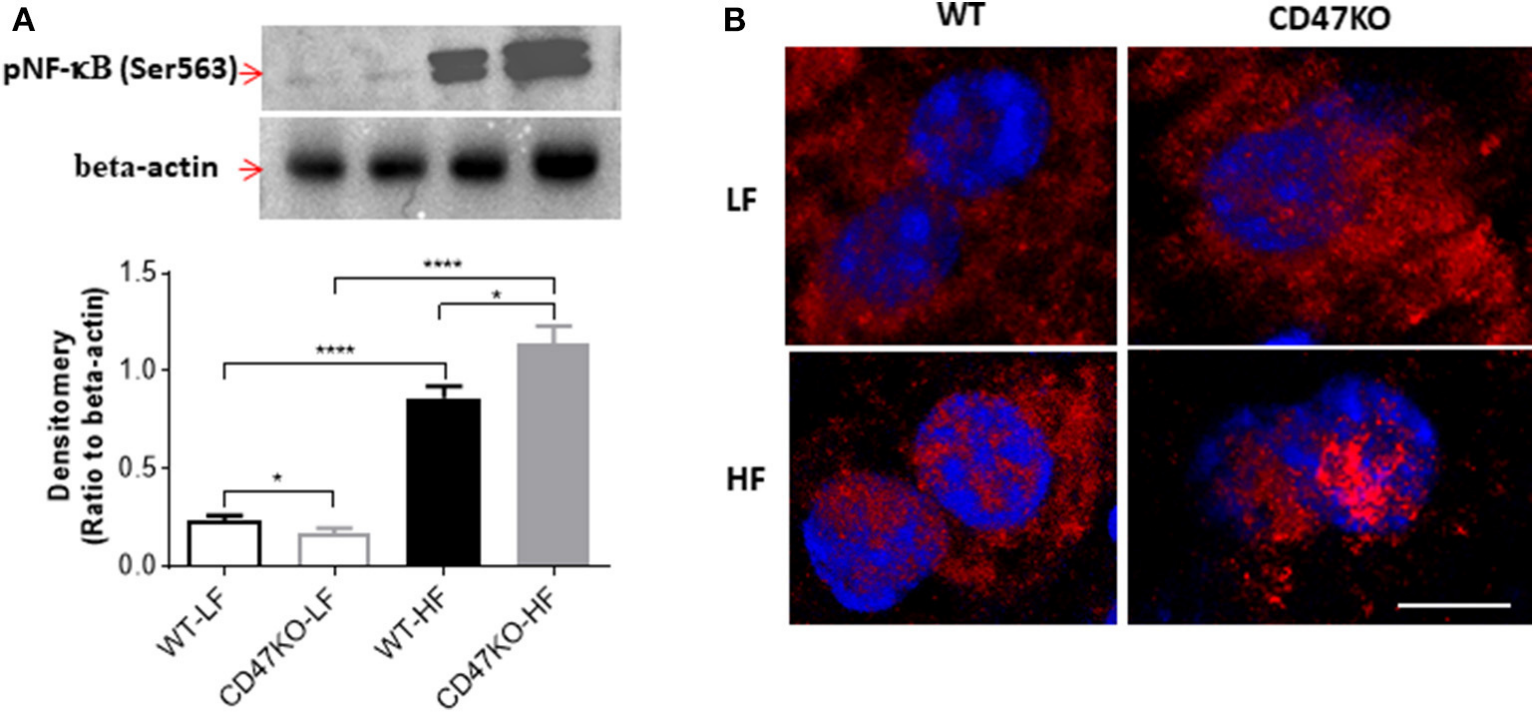
CD47 deficiency promotes HFD-induced nuclear factor-κB (NF-**κ**B) activation and translocation. **(A)** Immunoblot analysis of phospho-NF-**κ**B p65 (Ser536) protein in liver tissues from WT and CD47KO mice fed LFD or HFD (upper) and band density normalized to β-actin [lower; data are means (±SDs) of three independent experiments]. **(B)** Confocal images showing subcellular distribution of phosphorylated NF-**κ**B (pNF-**κ**B) (Ser536); pNF-**κ**B (Ser536); and nucleus were stained in red by AF594 and in blue by DAPI, respectively. The representative images from four independent samples are shown. Scale bar represents 10 μm. Data are presented as mean ± SD. ^*^p < 0.05 and ^****^P < 0.0001.

As a master regulator of inflammation, NF-κB activation promotes the secretion of inflammatory cytokines and is accompanied by chronic hepatic injury and the development of liver fibrosis (24). Thus, we next compared NF-κB activation in liver tissues from WT and CD47KO mice by measuring phosphorylation at Ser536 and nuclear translocation of NF-κB p65. Although HFD resulted in enhanced NF-κB p65 phosphorylation at Ser536 in both WT and CD47KO mice, the effect was significantly stronger in the latter group (Figure 6A). Furthermore, markedly increased accumulation of phosphorylated NF-κB p65 (Ser536) in the nucleus was detected in liver tissues from HFD-fed CD47KO mice (Figure 6B). Taken together, our results indicate that HFD induces more severe inflammatory responses in CD47KO than in WT mice.

### PPARα and SIRT1 Expression Increases Under LFD Feeding and Is Significantly Downregulated Under HFD Feeding in CD47KO Mice

**Figure 7:**
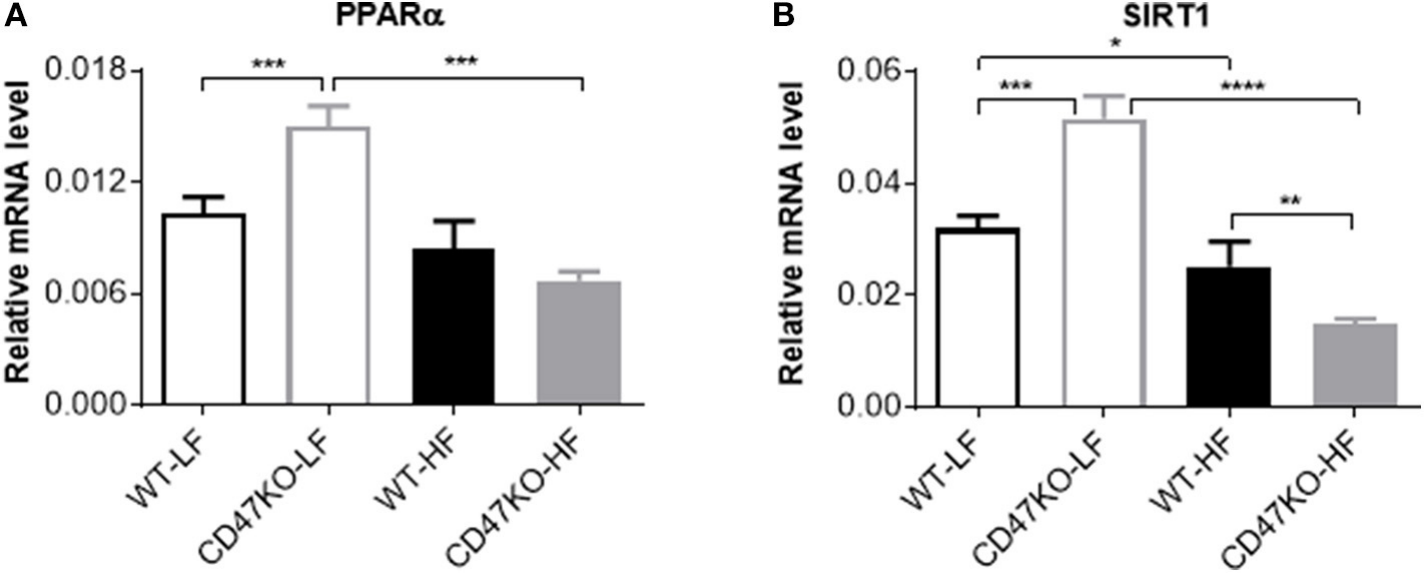
Distinct effects of CD47 deficiency on peroxisome proliferator activated receptor alpha (PPARα and sirtuin 1 (SIRT1) expression between mice fed LFD vs. HFD. Relative mRNA expression levels of PPARα **(A)** and SIRT1 **(B)** were quantified by real-time PCR and normalized to β-actin (*n* = 4 per group). Data are presented as mean ± SD. ^*^p < 0.05; ^**^p < 0.01; ^***^P < 0.001; and ^****^P < 0.0001.

We also compared the expression of PPARα and SIRT1 in liver tissues from WT and CD47KO mice fed LFD or HFD. PPARα, which can be activated by fatty acids, plays an important role in the regulation of hepatic lipid metabolism, and its activity is inversely correlated with NAFLD in both humans and mice (25). PPARα was also reported to inhibit inflammation via regulation of NF-κB activity (26, 27). SIRT1 inhibits lipogenesis through deacetylating and activating PPARα (28). Under LFD, significantly increased PPARα expression was seen in CD47KO mice compared to WT mice (Figure 7A). However, HFD-fed CD47KO mice showed significant downregulation of PPARα compared to LFD-fed CD47KO mice, while there was no significant downregulation of PPARα in HFD-fed WT mice compared to LFD-fed WT mice (Figure 7A). In line with this finding, SIRT1 expression in the liver was also significantly higher under LFD but more severely suppressed by HFD in CD47KO mice compared to WT mice (Figure 7B). These results indicate that CD47 deficiency may affect lipid metabolism under both LFD and HFD conditions. Since PPARα and SIRT1 also have anti-inflammatory activity, the more severely downregulated expression may also contribute to enhanced hepatic inflammation in HFD-fed CD47KO mice.

## Discussion

NASH involves a range of pathologic conditions in the liver, including steatosis, inflammation, hepatocyte injury, and fibrosis. In this study, we showed that CD47KO mice chronically fed HFD (for 40 weeks) developed severe hepatomegaly and hepatic injury but no subcutaneous fat accumulation compared to WT mice similarly fed HFD. Liver tissues from HFD-fed CD47KO mice showed significantly more lipid accumulation than liver tissues from HFD-fed WT mice, but both groups had comparable downregulation of apolipoproteins. In livers from HFD-fed CD47KO mice, we consistently found more pronounced collagen production and fibrosis, and the associated abnormally enhanced angiogenesis, compared to HFD-fed WT mice. Notably, HFD-fed CD47KO mice exhibited markedly greater proinflammatory responses and more significantly reduced expression of PPARα and SIRT1, which are both known to regulate lipid metabolism and inflammation (29–32), compared to similarly fed WT mice. These findings provide direct evidence for an important role of CD47 in the pathogenesis of NASH induced by a fatty diet.

Hepatic fibrosis is triggered by chronic liver injury following sustained inflammation. CD47 has been shown to play an important role in regulating macrophage activation and phagocytosis by interacting with SIRPα, an inhibitory receptor on innate immune cells. It has been shown that CD47/SIRPα is essential in controlling macrophage activation by proinflammatory cytokines (12) and allogeneic or xenogeneic cell stimuli (10, 13–15). Here we show that CD47 deficiency also promotes HFD-induced inflammatory responses in the liver, including enhanced proinflammatory cytokine production and the associated activation of NF-κB and secretion of the potent macrophage chemoattractant chemokine CCL2. Furthermore, increased HFD-induced inflammation in CD47KO mice was correlated with the severity of hepatosteatosis and fibrosis, supporting the previously noted role of inflammation in the development of NAFLD.

In the present study, we found that PPARα and SIRT1 expression levels in the liver of CD47KO mice were significantly elevated and inhibited under LFD and HFD conditions, respectively, compared to WT mice. Previous studies have shown that fatty acids play an important role in the activation and stimulation of PPARα and SIRT1 expression, but NAFLD is often associated with failed/reduced expression of these genes in both humans and mice (28, 33). Thus, it is possible that the reduced expression in HFD-fed CD47KO mice was due to severe steatosis similar to that found in patients with established NAFLD. Although further studies are needed to unveil the mechanisms behind the altered expression of PPARα and SIRT1 in the absence of CD47, our results suggest that CD47 may also be involved in the pathogenesis of HFD-induced NASH through its role in regulating lipid metabolism. In addition to being potent regulators of lipid metabolism, PPARα and SIRT1 have also been reported to inhibit proinflammatory responses. SIRT1 has a pivotal role in the regulation of PPARα activation and antagonistic inhibition of NF-κB in the liver (34, 35). Furthermore, PPARα activation downregulates IL-1β and TNFα in the liver via inhibition of NF-κB and suppresses hepatosteatosis via upregulation of fatty acid oxidation gene expression in rodent NAFLD models (31, 32). Here we found that the levels of PPARα and SIRT1 expression were inversely correlated with NF-κB activity in HFD-fed mice, which is consistent with the previously reported antagonistic crosstalk between NF-κB and SIRT1 (36, 37). Taken together, these observations indicate that the more severe inhibition of PPARα and SIRT1 expression may contribute to the augmented hepatic inflammation in HFD-fed CD47KO mice.

HFD induces a marked increase in circulating TSP1 in the early stages of HFD challenge, and TSP1 deficiency protects mice from HFD-induced weight gain and adipocyte hypertrophy (38). Thus, it is likely that the loss of TSP1/CD47 signaling also contributes to the reduced weight gain and reduced subcutaneous fat accumulation observed in CD47KO mice fed HFD. However, the current study cannot mechanistically explain the contradictory findings in subcutaneous vs. liver fat metabolism in CD47KO mice under HFD. A previous report showed that CD47 deficiency protects against fat accumulation in both subcutaneous tissue and liver, in mice fed HFD for a shorter period of time (16 weeks) (16). It is possible that the effect of CD47 deficiency may differ depending on the conditions of the tissue microenvironment. These differences might include the proclivity of tissue-resident cells to cause and/or respond to inflammation and the strength of proinflammatory stimuli, and the degree of tissue damage. These factors are expected to be different depending on the tissue and the time after HFD feeding.

TSP1 is a potent inhibitor of angiogenesis (39), and its anti-angiogenic activity can be mediated by its receptor CD47 (40–42). In line with these observations, CD47 deficiency in endothelial cells (ECs) was found to enable ECs to gain stem cell characteristics (43), and to improve angiogenic function by promoting proliferation and attenuating replicative senescence of ECs (40). TSP1/CD47 signaling also inhibits VEGF2 phosphorylation in ECs (44), and CD47 deficiency enhances neovascularization in tumors (45). Thus, abrogation of TSP1/CD47 signaling is likely to be a key mechanism for the observed upregulation of VEGFR and the abnormally enhanced angiogenesis in the livers of HFD-fed CD47KO mice.

In conclusion, this study provides direct evidence that CD47, a ligand of SIRPα, an inhibitory receptor on myeloid innate immune cells, plays a significant role in the pathogenesis of HFD-induced hepatosteatosis and fibrosis. Our data also suggest that the role of CD47 in NASH induced by chronic HFD consumption is mediated by its effects on hepatic inflammation and lipid metabolism.

## Data Availability Statement

The datasets generated for this study are available on request to the corresponding author.

## Ethics Statement

This animal study was reviewed and approved by Subcommittee on Research Animal Care of the First Hospital of Jilin University.

## Author Contributions

H-CT and K-XC designed, performed, and analyzed data from most of the experiments. XW, BC, and W-OZ performed histology and immunofluorescence analysis. K-XC, YZ, and Y-GY conceived, designed, and supervised all studies. K-XC and Y-GY wrote the manuscript. All authors read and approved the final manuscript.

### Conflict of Interest

The authors declare that the research was conducted in the absence of any commercial or financial relationships that could be construed as a potential conflict of interest.
