# Supplementary material for: CD47 Deficiency in Mice Exacerbates Chronic Fatty Diet-Induced Steatohepatitis Through Its Role in Regulating Hepatic Inflammation and Lipid Metabolism
Source: Front Immunol. 2020 Feb 25;11:148. doi: 10.3389/fimmu.2020.00148 (PMC7052326; doi:10.3389/fimmu.2020.00148)
Supplement: Supplementary file 1 [file Data_Sheet_1.PDF]

Supplementary Data

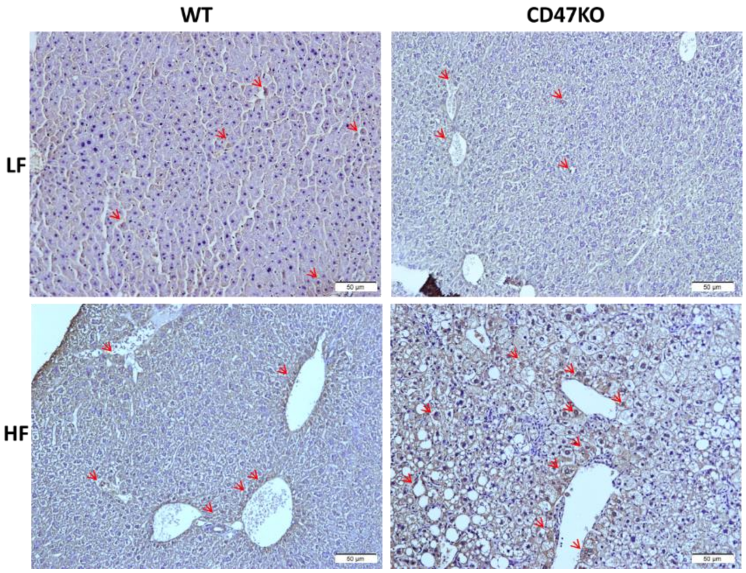

Figure S1. Liver sections were IHC stained for TGF-β. Three samples per group were examined, and representative images are shown (Scale bar represents 50 μm).

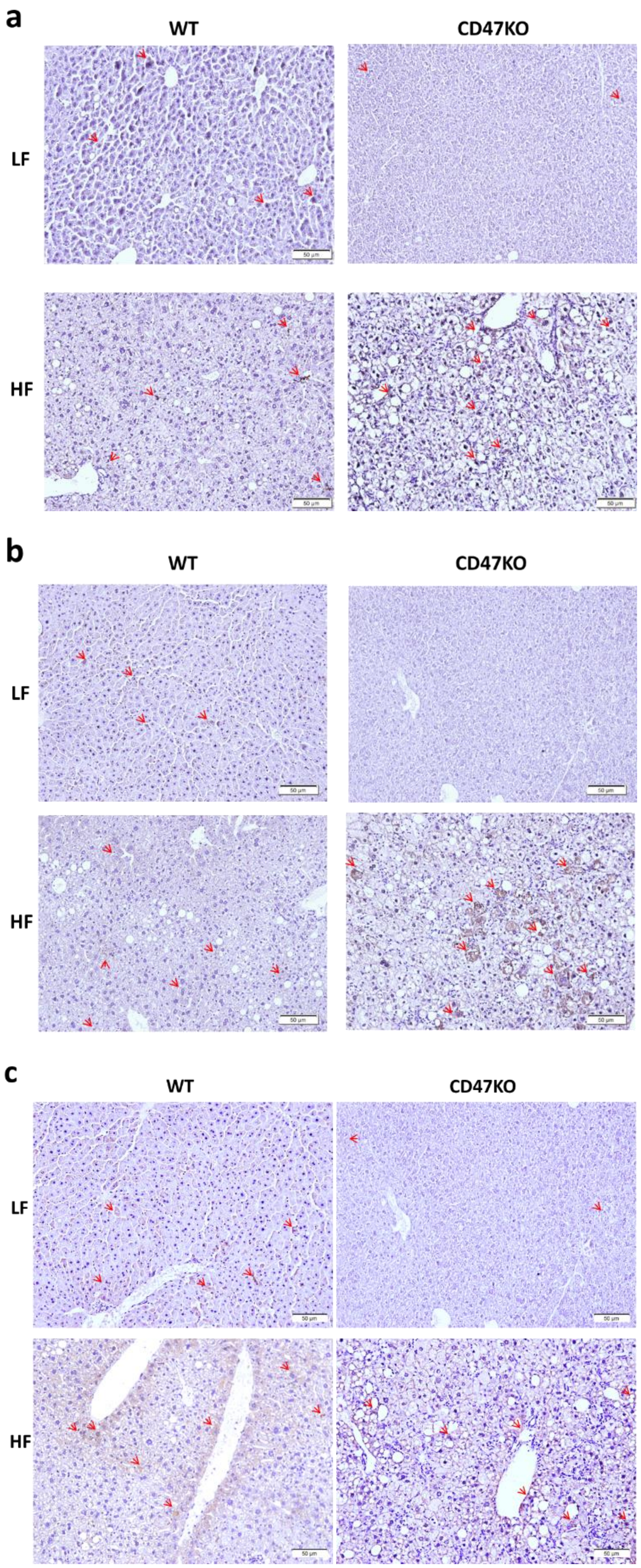

Figure S2. Liver sections were IHC stained for TGF-β (a), IL-6 (b) and IL-10 (c). Three samples per group were examined, and representative images are shown (Scale bar represents 50 μm).

Table S1. High fat diet formulation
(http://www.jsxtsw.com/wp-content/uploads/2019/05/image-1.png)

Class description	Ingredient		Grams
Protein	Casein, Lactic, 30 Mesh		200.00 g
Protein	Cystine, L		3.00 g
Carbohydrate	Sucrose, Fine Granulated		176.80 g
Carbohydrate	Lodex 10		100.00 g
Carbohydrate	Starch, Corn		72.80 g
Fiber	Solka Floc, FCC200		50.00 g
Fat	Lard		177.50 g
Fat	Soybean Oil, USP		25.00 g
Mineral	S10026B		50.00 g
Vitamin	Choline Bitartrate		2.00 g
Vitamin	V10001C		1.00 g
Dye	Dye, Red FD&C #40, Alum. Lake 35-42%		0.05 g
		Total:	858.15 g
